# Multiple intracranial arterial stenosis influences the long-term prognosis of symptomatic middle cerebral artery occlusion

**DOI:** 10.1186/s12883-015-0326-0

**Published:** 2015-04-29

**Authors:** Lili Tian, Xuanye Yue, Gangming Xi, Youmeng Wang, Zongyou Li, Ying Zhou, Xiaobing Fan

**Affiliations:** Department of Neurology, Jinling Hospital, Nanjing University School of Medicine, 305 East Zhongshan Road, Nanjing, 210002 Jiangsu Province China; Department of Neurology, The People’s Hospital of Fuyang City, 63 Luci Road, Fuyang, 236010 Anhui Province China; Department of Neurology, Department of Neurology and Tianjin Neurological Institute, Tianjin Medical University General Hospital, Tianjin, 300052 China; Department of Neurology, Shanghai Clinical Center, Chinese Academy of Sciences/Shanghai Xuhui Central Hospital, 966 Middle Huaihai, Shanghai, 200030 China

**Keywords:** Prognosis, Stroke, Middle cerebral artery, Occlusion

## Abstract

**Background:**

Large intracranial occlusive vascular disease is a major contributor to the incidence of stroke worldwide, especially when it involves the middle cerebral artery (MCA). The data on the prognosis of symptomatic atherosclerotic MCA occlusions (MCAO) with concomitant intracranial arterial disease (MCAO-AIS) are limited. MCAO-AIS may reflect the extent of the atherosclerotic intracranial disease, we hypotheses that coexisting intracranial arterial disease influenced the prognosis of MCAO.

**Methods:**

Patients having survived at least one month after the initial ischemic stroke who suffered from atherosclerotic occlusion of the MCA were enrolled. According to their concomitant atherosclerotic intracranial arterial disease, the patients were assigned to one of two groups: the MCAO or the MCAO-AIS. All of the patients’ cerebrovascular risk factors were recorded. Recurrent ischemic stroke and death were the end-point events during the follow-up.

**Results:**

A total of 232 patients (mean age 57.68 ± 9.50 years; 69% male) were analyzed. The mean follow-up time was 17.65 months. The end-point events occurred in 35 (15.09%) patients, resulting in an annual rate of 10.26%. The presence of MCAO- AIS was an independent risk factor associated with the patient’s prognosis in the cohort (OR = 3.426, 95% CI 1.261 to 9.308; *p* = 0.016), as well as gender and diabetes mellitus. The MCAO-AIS were more likely to experience ipsilateral ischemic strokes, but the difference was not statistically significant.

**Conclusion:**

Concomitant intracranial arterial disease may influence the prognosis of patients with atherosclerotic MCAO. The result warrants further research in larger sample population.

## Background

Intracranial atherosclerotic occlusion or stenosis is an important cause of ischemic stroke in both Asians and African Americans [1-3]. The middle cerebral artery (MCA) is one of the most common sites involved in symptomatic intracranial atherosclerosis. Previous studies have reported that the annual risk of stroke in patients with symptomatic MCA stenosis is 4% ~ 15% [4-7]. Although the prognosis of patients with symptomatic and asymptomatic MCA stenosis has been studied previously, few studies have reported data on patients with a middle cerebral artery occlusion (MCAO). Furthermore, the previous studies have reported that the recurrence of stroke in patients with MCAO did not differ from stroke recurrence with severe MCA stenosis [5,6,8,9]. These studies included a very small number of patients. Therefore, greater knowledge of the natural course or cerebrovascular events of MCAO disease is need, especially considering its few treatment options.

Previous studies have reported that diabetes mellitus, stroke symptoms, and gender may influence the prognosis of patients with symptomatic MCAO [7,9,10]. However, there is little in the literature on MCAO with concomitant intracranial arterial disease (MCAO-AIS). The rate of MCAO-AIS in patients with symptomatic stenosis is high, approximately 18.9% to 27.3% [11]. MCAO-AIS may suggest a generalized atherosclerotic process, which may portend a poor prognosis [12]. The number of coexisting intracranial arterial disease (AIS) may also reflect the extent of the atherosclerotic intracranial disease. Considering the points above, we hypothesized that coexisting AIS may influenced the prognosis of patients with symptomatic MCAO.

We therefore conducted this retrospective analysis to evaluate the further progression of MCAO-AIS and its potential risk factors for stroke recurrence and mortality in these patients.

## Methods

### Subjects

We consecutively recruited patients who were admitted to our hospital and had cerebral angiographic records from January 2006 to January 2010. The data were documented prospectively in the Nanjing Stroke Registry Program. Patients were included if they met the following criteria: 1) occlusion in the M1 segment of the MCA that was proven by magnetic resonance angiography (MRA) or cerebral digital subtraction angiography (DSA), 2) an initial ischemic stroke or transient ischemic stroke (TIA) within the occluded MCA distribution territory, 3) at least one risk factor of atherosclerosis, 4) survival for at least one month after the onset, and 5) an age greater than 40 years old.

We excluded: 1) patients with a history of stroke or TIA, 2) those who died during one month after the one of symptoms, 3) patients with atrial fibrillation (AF) which included chronic or paroxysmal AF. Paroxysmal atrial fibrillation was defined as episodic AF on an electrocardiogram, or transient AF (<24 hours) on a 24-hour ambulatory electrocardiographic recording. 4) patients with MCAO due to non-atherosclerotic etiology such as vasculitis, vasospasm, dissection, aneurysm, basilar meningitis, or Moyamoya disease, 5) patients with recanalization of the occluded MCA after endovascular therapy, 6) and patients who presented with cancer, severe liver disease, renal failure, or hematological disease.

In this study, all patients were performed MRA. One hundred twenty-eight patients were performed DSA. The magnetic resonance imaging was performed with a 1.5-T superconductive magnetic resonance system, and all of the MRA examinations were obtained using three-dimensional (3D) gadolinium (Gd)-enhanced technique. A DSA was performed in all patients in whom MCA occlusion was suspected from the MRA. The DSA were performed after the vital signs were stabilized. The location and severity of the stenosis was determined using an MRA or DSA and the Warfarin-Aspirin Symptomatic Intracranial Disease (WASID) trial criteria in all of the patients [8,13]. The absence of distal filling of the MCA on MRA or DSA could be regarded as MCA occlusion [14]. The vessels with >50% stenosis or occlusions presence were categorized as intracranial stenosis. By definition, patients with “flow gaps” were considered to have an AIS (defined as 50% to 99% stenosis) [11]. If there was more than one intracranial disease, the most severe degree of stenosis was measured. According to the AIS, all of the patients were assigned to one of two groups: MCAO or MCAO-AIS group. The cerebrovascular vessels were assessed by two experienced investigators who used either an MRA or DSA at the patient’s entry into the study and at each follow-up visit and who were blinded to the other’s findings. Patients were excluded if the MCA occlusion was caused by dissection or radiation therapy.

This study was approved by the ethics committee of Jinling Hospital, and written informed consent was obtained from all patients.

### Clinical backgrounds and characteristics

We reviewed the patients’ baseline demographic factors and the medical records. The main risk factors for MCAO were also recorded, such as hypertension (use of antihypertensive agents, systolic blood pressure >140 mmHg or diastolic blood pressure >90 mmHg before or at least 2 weeks after the stroke’s onset), diabetes mellitus (use of hypoglycemic agents, a fasting blood glucose >7.0 mmol/L or a postprandial blood glucose after 2 h >11.1 mmol/L), hyperlipidemia (use of antihyperlipidemic medication, or a total serum cholesterol level > 6.0 mmol/L or LDL cholesterol concentration 3.63 mmol/l), a history of coronary heart disease, cigarette smoking (at least 10 cigarettes per day during the past 5 years or more), and alcohol intake (consumed regularly more than 300 g alcohol/week). Previous use of antiplatelet agents, anticoagulants, or statins was also evaluated.

### Follow-up

We followed the patients who survived the acute phase of stroke discharged after 1, 3, 6, 12 months by interviews or telephone to determine whether any events had occurred. Asymptomatic patients had subsequent yearly follow-up visits. The patients were asked whether they had experienced any sudden episode of weakness, blindness, numbness, speech difficulty and any other new symptoms and documented in Nanjing Stroke Registry Program. If a stroke was suspected, the patients were invited to our Neurology Clinic and were evaluated by study neurologists who were blinded to the treatment assignment and also underwent brain computerized tomography (CT) or magnetic resonance imaging (MRI). The interviewers were blinded to the patient’s group allocation. The composite outcome of the stroke recurrence or any-cause death was evaluated during the follow-up period to elucidate the long-term prognosis of symptomatic MCAO patients.

An ischemic stroke was defined as a new focal neurological deficit of sudden onset ≥ 24 hours in duration that, was not associated with a hemorrhage on neuroimaging. An ipsilateral stroke was considered to have occurred in the same location of the symptomatic artery when the neurological signs correlated with a new infarct on a CT or MRI in an area of the brain supplied by that artery.

### Statistical analysis

The statistical analysis was performed with SPSS version 13.0 software (Chicago, Illinois, USA). A chi-square or Fisher’s exact test was used to compare the rate and proportions of the discrete variables, and Student’s-t tests were performed for the continuous variables. We used the Cox proportional hazards models for the univariate and multivariate analyses to identify the potential factors associated with stroke recurrence or any-cause death. A Kaplan-Meier curve was generated for the cumulative probabilities of stroke recurrence and the composite outcome, and the two groups (MCAO and MCAO-AIS) were compared using the log-rank test. The patients who were lost to follow-up were censored at the last contact date in the analysis. The results were expressed as the adjusted odds ratio (OR) and corresponding 95% confidence intervals (95% CI). For all statistical tests, values are 2-sided and *p* < 0.05 was considered to indicate statistically significant differences.

## Results

During the study, 3460 patients were enrolled from the databank. Three hundreds and six (8.8%) patients were recruited, all of them were first-ever stroke. Among them, 35 patients died in the acute phase (11.4%) and, 39 patients (14.39%) underwent a surgical revascularization procedure and were excluded. Therefore, we included 232 first-ever stroke patients with complete occlusion of the MCA in this study, and followed them for an average of 17.65 ± 10.81 months (range, 1 to 39 months). Seven (3.02%) patients were lost during the follow-up. The mean age was 57.68 ± 9.50 years, and 69% of the patients were male. All of the patients were continuously treated with both antiplatelet agents and statins if the patients had not any adverse events. Eighty-four patients were well controlled their lipid level (total cholesterol (TC) <3.6 mmol/L, LDL-C < 1.8 mmol/l). The distribution of the baseline characteristics and the composite outcome (stroke recurrence or any-cause death) is listed in Table 1.

**Table 1:** **Comparison of demographic and clinical characteristics between MCAO and MCAO-AIS**

	**MCAO (n = 84)**	**MCAO-AIS (n = 148)**	***P*** **value**
Age, years	55.58 ± 9.48	58.85 ± 9.35	0.666
Gender (Male)	59 (71.1%)	101 (67.8%)	0.603
Hypertension (Yes)	39 (47.0%)	97 (65.1%)	0.007
Diabetes mellitus (Yes)	18 (21.7%)	43 (28.9%)	0.234
Hyperlipidemia (Yes)	18(21.7%)	27 (18.1%)	0.510
Coronary artery disease (Yes)	17 (20.5%)	27 (18.1%)	0.660
Smoking (Yes)	42 (50.6%)	68 (45.6%)	0.468
Drinks alcohol (Yes)	27 (32.5%)	40 (26.8%)	0.360
Composite outcome (Yes)	5 (6.0%)	30 (20.1%)	0.004

Notes: MCAO, middle cerebral artery occlusion. MCAO-AIS, middle cerebral artery occlusion with concomitant intracranial arterial disease.

The total patients in MCAO-AIS is 149 (64.22%). The patients in MCAO-AIS were more likely to have high blood pressure at enrollment (65.1% versus 47.0%, *p* = 0.007), and a poor composite outcome (20.1% versus 6.0%, *p* = 0.004) than their counterparts MCAO (Table 1).

### Rate of stroke recurrence or any-cause death

During the follow-up period, 11 (4.74%) of the 232 patients died (three women (1.29%) and eight men (3.45%)). Three patients (1.29%) died from the initial stroke, two (0.86%) died from a recurrent stroke, one (0.43%) died from cardiovascular disease, and five (2.16%) died from pulmonary causes (pneumonia or pulmonary embolisms).

**Figure 1:**
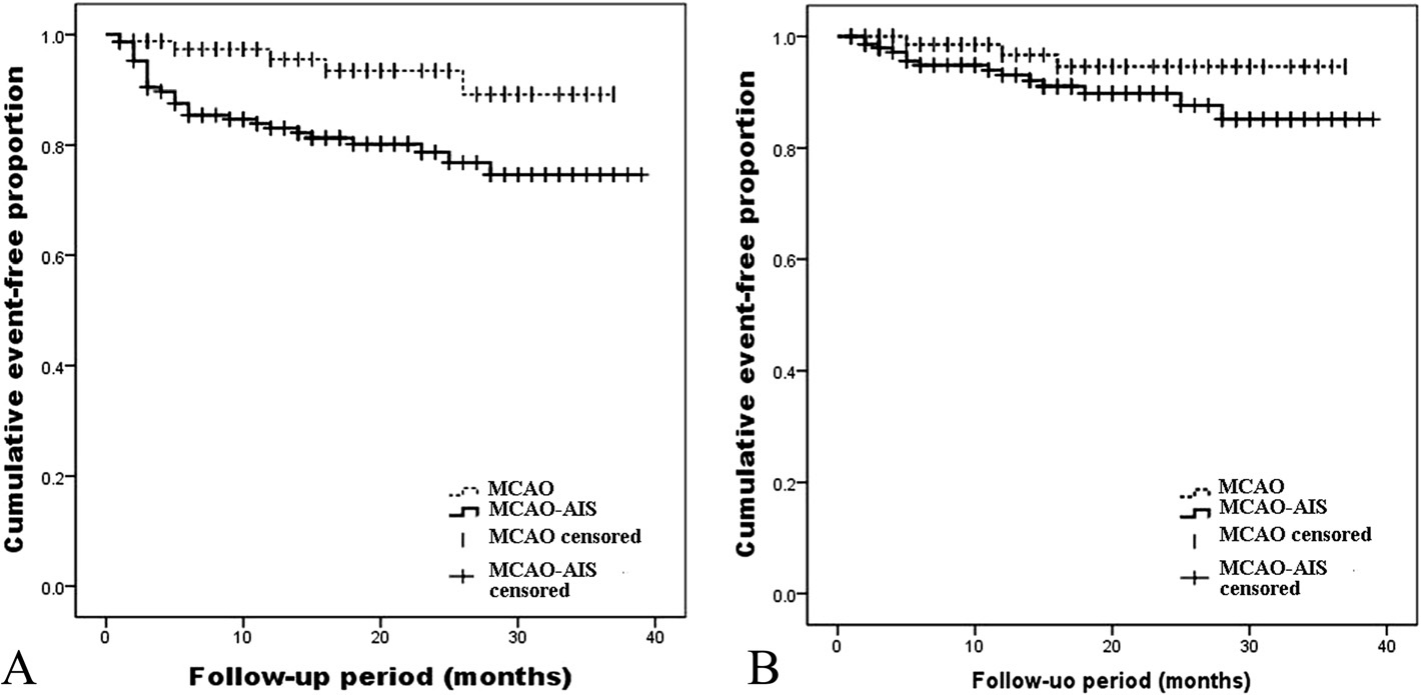
Comparison of the survival curves estimated with the Kaplan-Meier model between the MCAO and MCAO-AIS groups (using the log-rank test), showing the cumulative event-free proportion of the total composite outcome **(A)** and ipsilateral ischemic stroke recurrence **(B)** during the observation period.

We documented 35 (15.09%) composite outcomes during the observation. The Kaplan-Meier curves showed that a significantly lower proportion of patients in the MCAO-AIS group survived without a composite outcome when compared with the MCAO group (79.90% vs. 94.00%, *p* = 0.009, Figure 1A). The cumulative stroke recurrence or any-cause death rate was 4.80% at one year, 21.30% at two years, and 25.40% at three years in the MCAO-AIS group, compared with 4.5%, 6.60%, and 10.80%, respectively, in the MCAO group.

### Risk factors of recurrent stroke or death

The variables that were potentially associated with the MCAO prognosis are listed in Table 2. The univariate analysis revealed that hypertension (OR = 3.715, 95% CI 1.541 to 8.958), diabetes mellitus (OR = 3.614, 95% CI 1.858 to 7.032), and the coexistence of AIS (OR = 3.265, 95% CI 1.267 to 8.416) were associated with the occurrence of stroke or any-cause death. Other stroke risk factors, such as hypercholesterolemia, smoking, and a family history of stroke, were not found to be correlated with stroke occurrence or death. These variables were included in the multivariate analysis of the Cox proportional hazard model. Then we found that the coexistence of AIS remained an independent predictor of symptomatic MCAO progression (OR = 3.426, 95% CI 1.261 to 9.308; *p* = 0.016). Moreover, diabetes mellitus was related to the recurrence of both overall (*p* < 0.01) and ipsilateral ischemic vascular events (*p* < 0.01).

**Table 2:** **Potential risk factors of cerebrovascular events and any-cause death of patients with symptomatic MCAO**

**Risk factors**	**Univariate OR**	**Multivariate OR**
	**(** ***P*** **value)**	**(** ***P*** **value)**
Age, years	1.010 (0.573)	0.980 (0.337)
Gender (Male)	0.562 (0.092)	0.444 (0.041)
Hypertension	3.715 (0.003)	2.465 (0.062)
Diabetes mellitus	3.614 (0.001)	2.842 (0.003)
Hyperlipidemia	1.710 (0.168)	2.065 (0.075)
Coronary artery disease	1.927 (0.080)	1.664 (0.186)
Smoking	1.067 (0.848)	1.427 (0.405)
Drinks alcohol	0.985 (0.967)	1.277 (0.599)
Coexisting AIS	3.265 (0.014)	3.426 (0.016)

Notes: MCAO, middle cerebral artery occlusion. OR, odds ratio.

Considering that the presence of MCAO-AIS showed a significant difference for the composite outcomes (*p* = 0.016), we further analyzed this subgroup, as shown in Table 3. Hypertension (*p* = 0.016), diabetes mellitus (*p* = 0.007), and coronary artery disease (*p* = 0.033) were significantly associated with stroke recurrences or any-cause death in the univariate analysis. These 3 variables were included in the logistic regression model. Diabetes mellitus was the only factor independently associated with the recurrence of cerebral vascular events or any-cause death during the follow-up (OR = 2.282, 95% CI 1.089 to 4.782; *p* = 0.029). The Kaplan-Meier curves showed that a significantly lower proportion of patients presenting with AIS survived free of a stroke recurrence or any-cause death compared with the MCAO group (Figure 1A). However, we refrained from further multivariable analysis of the patients without concomitant AIS, because of the relatively small number of composite outcomes.

**Table 3:** **Univariate and Multivariate Model of outcome for symptomatic MCAO-AIS**

**Risk factors**	**Univariate OR**	**Multivariate OR**
	**(** ***P*** **value)**	**(** ***P*** **value)**
Age, years	0.996 (0.854)	0.978 (0.314)
Gender (Male)	0.573 (0.131)	0.465 (0.082)
Hypertension	3.635 (0.016)	3.011 (0.051)
Diabetes mellitus	2.685 (0.007)	2.282 (0.029)
Hyperlipidemia	1.773 (0.188)	2.102 (0.102)
Coronary artery disease	2.349 (0.033)	1.792 (0.157)
Smoking	0.897 (0.768)	1.220 (0.688)
Drinks alcohol	0.976 (0.953)	1.443 (0.486)

MCAO-AIS, middle cerebral artery occlusion with concomitant intracranial arterial disease. OR, odds ratio.

### Risk of an ipsilateral cerebral ischemic event

A new ischemic vascular event was noted in 22 patients (13 strokes and 9 TIAs), resulting in an overall annual rate of 6.45%. Seventeen events (77.27%) occurred in the location of the symptomatic occlusive MCA. Strokes located beyond the symptomatic MCAO occurred in 5 (22.73%) of the 22 patients. Of these 17 ipsilateral events, 11 were ischemic strokes (annual rate 3.22%) and 6 were TIAs (annual rate 1.76%). Among the patients in MCAO-AIS, 14 subjects experienced ipsilateral cerebrovascular events (10 strokes, 4 TIAs), with an occurrence rate of 7.00% in the first year, 10.2% in the second year, and 14.80% in the third year, compared with 3.30%, 5.40%, and 5.40% in the MCAO group respectively (1 stroke, 2 TIAs). The patients in MCAO-AIS tended to have ipsilateral ischemic strokes more frequently than those without AIS (9.40% vs. 3.60%), but this trend was not statistically significant in the Kaplan-Meier plots (*p* = 0.131) (Figure 1B).

Both hypertension (*p* = 0.017) and diabetes mellitus (*p* = 0.000) were significantly associated with ipsilateral ischemic vascular event in the univariate analysis (Table 4). Both of these variables were included in the logistic regression model, and diabetes mellitus remained an independent predictor for the occurrence of ipsilateral ischemic strokes and TIAs (OR = 5.671, 95% CI 1.926 to 16.694; *p* = 0.002).

**Table 4:** **Potential predictors of ipsilateral stroke recurrence**

**Risk factors**	**Univariate OR**	**Multivariate OR**
	**(** ***P*** **value)**	**(** ***P*** **value)**
Age, years	1.027 (0.321)	0.995 (0.880)
Gender (Male)	0.595 (0.292)	0.358 (0.078)
Hypertension	6.047 (0.017)	3.318 (0.135)
Diabetes mellitus	7.427 (0.000)	5.671 (0.002)
Hyperlipidemia	1.651 (0.383)	2.087 (0.248)
Coronary artery disease	2.698 (0.051)	2.407 (0.101)
Smoking	1.610 (0.334)	2.007 (0.253)
Drinks alcohol	1.317 (0.588)	1.522 (0.524)
Coexisting AIS	2.523 (0.146)	2.948 (0.132)

Notes: AIS, intracranial arterial disease. OR, odds ratio.

## Discussion

This follow-up study provides evidence about the dynamic nature of symptomatic MCA atherosclerotic occlusive disease with coexisting AIS. We found that the prevalence of concomitant AIS (50% to 99%) in our cohort of patients with symptomatic MCAO was high (63.79%). Our data suggests that the rate of stroke recurrence or any-cause death in MCAO-AIS is much higher than in MCAO (*p* < 0.01).

Despite the widespread availability of valid diagnostic methods, there is little reported in the literature on the prognostic markers following an MCA occlusion. Our data demonstrated that diabetes, the systolic blood pressure at enrollment, and MCAO-AIS were statistically significant, modifiable risk factors for the prognosis of patients with MCAO. Diabetes mellitus is a well-known risk factor for both small and large vessel diseases. Previous studies have identified diabetes mellitus as a risk factor for recurrent vascular events or death in patients with predominantly intracranial atherosclerosis [9,10]. We also identified the presence of diabetes mellitus as a potential independent potential predictor of the long-term outcome, stroke recurrence, or any-cause death. The poor control of blood pressure appeared to be associated with a higher risk of a subsequent stroke [15]. In addition, the prevalence of coexisting asymptomatic intracranial stenosis in our cohort of patients with MCAO was 63.79% and was an independent risk factor for the prognosis of MCAO stroke patients. Collaterals also should be considered in the MCAO-AIS patients, as they can be observed to varying extents in intracranial atherosclerosis, whereas the presence of any collateral in moderate stenosis patients was an ominous predictor of stroke [16]. Individuals with robust collaterals may be at risk for stroke, although to a lesser extent than those without collaterals. Arenillas and his colleagues reported that the number of coexisting asymptomatic stenoses showed a trend toward a higher recurrence rate [5]. Others have reported that several variables, such as severe stenosis, stroke symptoms, and being female, were predictors of a subsequent stroke in the location of the stenotic artery among patients with AIS [4]. However, there is relatively little information on the relationship between the risk factors and the recurrence of vascular events in patients with an intracranial artery stenosis or occlusion, which remains to be further studied.

Although the mechanisms of ischemic stroke in intracranial atherosclerosis remain largely unexplored, the diminished flow beyond a stenosis may cause a stroke due to hypoperfusion or the impaired washout of the thromboemboli. Arterial occlusion due to the progressive atherosclerotic stenosis of an intracranial segment may allow robust collaterals to develop over time, although the relationship of these collaterals to the cerebral blood flow and clinical symptomatology remains unclear. Collaterals could radically decrease the risk of recurrent stroke in traditionally defined chronic disorders such as intracranial atherosclerosis and moyamoya disease. MCAO-AIS indicate poor collaterals, and the reduced antegrade of the forward flow beyond the lesion may cause a stroke due to hypoperfusion [17]. The cases enrolled in the WASID trial were symptomatic with respect to a particular arterial lesion, but the role of multifocal disease may provide an opportunity to correlate collaterals with concomitant asymptomatic lesions, as well as with the symptomatic intracranial atherosclerotic lesion [11]. Therefore, collaterals and the functional demonstration of flow impairment may be more informative than the isolated anatomical measures of maximal stenosis or length. Moreover, decreased perfusion reduces the washout and clearance of emboli [18]. In addition, the presence of MCAO-AIS may indicate that the arterial atherosclerosis is more severe and extensive, identifying an unstable, evolving plaque [16]. Then, the ischemia may be incited due to artery-to-artery emboli. However, our measures of the maximal degree of stenosis are only rudimentary measures of the plaque architecture that influences the hemodynamic parameters. The lesion length, luminal irregularity, vessel tortuosity, and other factors beyond these parameters may cause hemodynamic impairment or thromboembolic potential.

The annual rate of cerebrovascular events in our study was 6.45%, which was much lower than in the WASID trial [19]. The mechanism of the low stroke risk in the patients surviving occlusions in the M1 segment of the MCA is uncertain, and may be attributable to the following. First, nearly all of the included subjects used antiplatelet therapy and statins. Previous studies have demonstrated that statins provide beneficial effects for the long-term functional outcomes in cerebral ischemia [20]. The pleiotropic effects of statins, which are beyond lipid lowering, include the modification of endothelial function, inflammatory responses, plaque vulnerability, and thrombus formation [21,22]. Second, our subjects were survivors of MCAO, which suggests that they may have good collaterals. Collaterals have had a dramatic role in averting stroke when the degree of arterial stenosis was severe [16]. A study demonstrated that patients with a decreased rCBF and rCVR resulting from an ICA or MCA occlusion have a higher risk of an ipsilateral or total ischemic stroke than those without such decreases. Although some of the patients with poor collaterals or the absence of collaterals died during the acute phases of their strokes and were excluded from this study, some of the patients with poor collaterals survived the acute phase of their strokes. Third, the MCAO patients were diagnosed by an MRA in previous studies, which may include severe stenosis of the MCA. The risk of a thrombus artery-to-artery embolism was higher in the patients with severe stenosis of the MCA compared with the MCA occlusion subjects. However, the cerebral anterior circulation is subject to more embolic infarctions and fewer incidents of severe intracranial atherosclerosis than the posterior/vertebrobasilar circulation [23].

There are some limitations in the present study. First, our study was a single-center study. The small number of cases and numerous risk factors may have led to the underestimation of the risk of MCAO due to a lack of statistical power. Future large- scale community or population-based studies are needed. Second, a conventional angiography was not performed in all of the MCAO patients because of its invasiveness, and the MRA does not have sufficient sensitivity and specificity values. Thus, the study may suffer from a selection bias. Third, as some of the cases were followed through telephone interviews for stroke recurrences, minor strokes may not have been observed.

## Conclusion

In conclusion, we demonstrated that the prevalence of concomitant AIS in our cohort of subjects with symptomatic MCAO was high and that the MCAO-AIS was an independent prognosis factor. The current results indicate that symptomatic MCAO patients with the presence of silent AIS may require intracranial angioplasty and stenting or intensive medical management to prevent recurrent strokes. According to the results of the Stenting vs. Aggressive Medical Management for Preventing Recurrent Stroke in Intracranial Stenosis (SAMMPRIS) trial, aggressive medical therapy was superior to percutaneous transluminal angioplasty and stenting with the use of the Wingspan stent system [24]. Therefore, we suggest that symptomatic MCAO-AIS patients should receive aggressive medical management, or stenting for severe stenosis if necessary [25]. However, the results of such management remain to be determined in future prospective trials.
